# Supplementary material for: Influencing factors and health risk assessment of microcystins in the Yongjiang river (China) by Monte Carlo simulation
Source: PeerJ. 2018 Nov 16;6:e5955. doi: 10.7717/peerj.5955 (PMC6241391; doi:10.7717/peerj.5955)
Supplement: Supplemental Information 1 — The river section includes three sampling points: Qingxiu District, Jiangnan District, and Yongning District. [file peerj-06-5955-s001.doc]

**Sampling registration form**

| Sampling location | In Qingxiu District, Jiangnan District, YongningDistrict, Nanning, China |
| --- | --- |
| Sampling time | Time: 11:00 to 14:00. The early part of the month is the 5th, the middle part is the 15th, and the late part is the 25th |
| Sampling staff | Chan-Chan Xiao , Mao-Jian Chen, Fan-Biao Mei , Xiang Fang. |

**Table 1 The concentration of microcystins from the beginning of March to December in Qingxiu District, Yongjiang River (μg/L)**

| Sampling time | EMCs | | | IMCs | | | T-RR | T-YR | T-LR | TMCs |
| --- | --- | --- | --- | --- | --- | --- | --- | --- | --- | --- |
|  | MC-RR | MC-YR | MC-LR | MC-RR | MC-YR | MC-LR |  |  |  |  |
| March | 0.0131 | 0.0046 | 0.0134 | 0.0204 | 0.0000 | 0.0342 | 0.0335 | 0.0046 | 0.0477 | 0.0857 |
| April | 0.0000 | 0.0945 | 0.0463 | 0.0242 | 0.0000 | 0.0121 | 0.0242 | 0.0945 | 0.0584 | 0.1771 |
| May | 0.0234 | 0.0000 | 0.0484 | 0.0044 | 0.0044 | 0.0564 | 0.0278 | 0.0044 | 0.1049 | 0.1371 |
| June | 0.0344 | 0.0000 | 0.1245 | 0.0257 | 0.0067 | 0.0000 | 0.0601 | 0.0067 | 0.1245 | 0.1912 |
| July | 0.1321 | 0.0084 | 0.1665 | 0.0000 | 0.0000 | 0.0801 | 0.1321 | 0.0084 | 0.2466 | 0.3871 |
| August | 0.1124 | 0.0543 | 0.0526 | 0.0852 | 0.0000 | 0.1137 | 0.1977 | 0.0543 | 0.1663 | 0.4183 |
| September | 0.0611 | 0.0221 | 0.2186 | 0.1191 | 0.0000 | 0.0000 | 0.1801 | 0.0221 | 0.2186 | 0.4208 |
| October | 0.0290 | 0.0000 | 0.0000 | 0.0071 | 0.1315 | 0.1022 | 0.0361 | 0.1315 | 0.1022 | 0.2699 |
| November | 0.0000 | 0.0423 | 0.1253 | 0.0945 | 0.0081 | 0.1144 | 0.0945 | 0.0504 | 0.2398 | 0.3847 |
| December | 0.0056 | 0.0000 | 0.0000 | 0.0000 | 0.0391 | 0.1897 | 0.0056 | 0.0391 | 0.1897 | 0.2344 |

The total content of MCs (TMCs) in water is the sum of the content of extracellular MCs (EMCs) and intracellular MCs (IMCs) dissolved in the water. The total RR(T-RR) is the sum of the intracellular MC-RR and extracellular MC-RR,T-YR; the total YR(T-YR) is the sum of the intracellular MC-YR and extracellular MC-YR; the total LR(T-LR) is the sum of the intracellular MC-LR and extracellular MC-LR(Table 2-9 is the same annotation).

**Table 2 The concentration of microcystins from the middle of March to December in Qingxiu District, Yongjiang River (μg/L)**

| Sampling time | EMCs | | | IMCs | | | T-RR | T-YR | T-LR | TMCs |
| --- | --- | --- | --- | --- | --- | --- | --- | --- | --- | --- |
|  | MC-RR | MC-YR | MC-LR | MC-RR | MC-YR | MC-LR |  |  |  |  |
| March | 0.0000 | 0.0209 | 0.0315 | 0.0022 | 0.0212 | 0.0000 | 0.0022 | 0.0421 | 0.0315 | 0.0759 |
| April | 0.0407 | 0.0000 | 0.0344 | 0.0000 | 0.0222 | 0.1132 | 0.0407 | 0.0222 | 0.1475 | 0.2104 |
| May | 0.0168 | 0.0056 | 0.0000 | 0.0024 | 0.0031 | 0.0034 | 0.0192 | 0.0087 | 0.0034 | 0.0313 |
| June | 0.0000 | 0.0253 | 0.0081 | 0.0144 | 0.0000 | 0.0368 | 0.0144 | 0.0253 | 0.0449 | 0.0846 |
| July | 0.0140 | 0.0157 | 0.0037 | 0.0054 | 0.0196 | 0.0054 | 0.0194 | 0.0353 | 0.0090 | 0.0637 |
| August | 0.0719 | 0.0108 | 0.0671 | 0.0033 | 0.0000 | 0.0565 | 0.0751 | 0.0108 | 0.1235 | 0.2095 |
| September | 0.0108 | 0.0000 | 0.0344 | 0.1122 | 0.0128 | 0.0455 | 0.1231 | 0.0128 | 0.0799 | 0.2158 |
| October | 0.1189 | 0.1112 | 0.1155 | 0.0000 | 0.0038 | 0.0000 | 0.1189 | 0.1150 | 0.1155 | 0.3493 |
| November | 0.0347 | 0.0000 | 0.0893 | 0.0120 | 0.0239 | 0.1261 | 0.0467 | 0.0239 | 0.2154 | 0.2860 |
| December | 0.1211 | 0.0900 | 0.0458 | 0.0069 | 0.0136 | 0.0272 | 0.1280 | 0.1036 | 0.0730 | 0.3046 |

**Table 3 The concentration of microcystins from the late March to December in Qingxiu District, Yongjiang River (μg/L)**

| Sampling time | EMCs | | | IMCs | | | T-RR | T-YR | T-LR | TMCs |
| --- | --- | --- | --- | --- | --- | --- | --- | --- | --- | --- |
|  | MC-RR | MC-YR | MC-LR | MC-RR | MC-YR | MC-LR |  |  |  |  |
| March | 0.0266 | 0.0196 | 0.0117 | 0.0376 | 0.0346 | 0.0295 | 0.0642 | 0.0542 | 0.0412 | 0.1596 |
| April | 0.0220 | 0.0092 | 0.0675 | 0.0000 | 0.0000 | 0.0000 | 0.0220 | 0.0092 | 0.0675 | 0.0988 |
| May | 0.0042 | 0.0000 | 0.0000 | 0.0033 | 0.0201 | 0.0350 | 0.0076 | 0.0201 | 0.0350 | 0.0626 |
| June | 0.0112 | 0.0000 | 0.0112 | 0.1029 | 0.0044 | 0.0000 | 0.1142 | 0.0044 | 0.0112 | 0.1298 |
| July | 0.0000 | 0.0091 | 0.0000 | 0.0709 | 0.0000 | 0.0122 | 0.0709 | 0.0091 | 0.0122 | 0.0922 |
| August | 0.0196 | 0.0000 | 0.0489 | 0.0234 | 0.0174 | 0.0439 | 0.0430 | 0.0174 | 0.0928 | 0.1532 |
| September | 0.0217 | 0.0000 | 0.0446 | 0.0000 | 0.0621 | 0.1212 | 0.0217 | 0.0621 | 0.1658 | 0.2496 |
| October | 0.1138 | 0.0169 | 0.1193 | 0.0145 | 0.0000 | 0.0117 | 0.1282 | 0.0169 | 0.1309 | 0.2760 |
| November | 0.0000 | 0.0762 | 0.0000 | 0.0432 | 0.0468 | 0.0366 | 0.0432 | 0.1230 | 0.0366 | 0.2028 |
| December | 0.0628 | 0.0416 | 0.0127 | 0.0726 | 0.0124 | 0.0233 | 0.1354 | 0.0540 | 0.0360 | 0.2255 |

**Table 4 The concentration of microcystins from the beginning of March to December in Jiangnan District, Yongjiang River (μg/L)**

| Sampling time | EMCs | | | IMCs | | | T-RR | T-YR | T-LR | TMCs |
| --- | --- | --- | --- | --- | --- | --- | --- | --- | --- | --- |
|  | MC-RR | MC-YR | MC-LR | MC-RR | MC-YR | MC-LR |  |  |  |  |
| March | 0.0345 | 0.0188 | 0.0112 | 0.0000 | 0.0472 | 0.0078 | 0.0345 | 0.0660 | 0.0190 | 0.1195 |
| April | 0.0000 | 0.0000 | 0.0084 | 0.0065 | 0.0041 | 0.0130 | 0.0065 | 0.0041 | 0.0214 | 0.0320 |
| May | 0.1040 | 0.1203 | 0.0000 | 0.0149 | 0.0000 | 0.0157 | 0.1189 | 0.1203 | 0.0157 | 0.2549 |
| June | 0.0750 | 0.0000 | 0.0137 | 0.0241 | 0.0125 | 0.0594 | 0.0991 | 0.0125 | 0.0730 | 0.1846 |
| July | 0.0000 | 0.0278 | 0.0000 | 0.0183 | 0.0472 | 0.0046 | 0.0183 | 0.0750 | 0.0046 | 0.0979 |
| August | 0.0461 | 0.0036 | 0.0167 | 0.0199 | 0.0000 | 0.0049 | 0.0660 | 0.0036 | 0.0216 | 0.0912 |
| September | 0.0045 | 0.0000 | 0.0608 | 0.1478 | 0.0092 | 0.0000 | 0.1522 | 0.0092 | 0.0608 | 0.2223 |
| October | 0.2035 | 0.1081 | 0.0406 | 0.0000 | 0.0000 | 0.0046 | 0.2035 | 0.1081 | 0.0452 | 0.3568 |
| November | 0.0942 | 0.0000 | 0.0264 | 0.0675 | 0.0391 | 0.0000 | 0.1616 | 0.0391 | 0.0264 | 0.2271 |
| December | 0.0214 | 0.0000 | 0.0155 | 0.0000 | 0.0065 | 0.0148 | 0.0214 | 0.0065 | 0.0304 | 0.0583 |

**Table 5 The concentration of microcystins from the middle of March to December in Jiangnan District, Yongjiang River (μg/L)**

| Sampling time | EMCs | | | IMCs | | | T-RR | T-YR | T-LR | TMCs |
| --- | --- | --- | --- | --- | --- | --- | --- | --- | --- | --- |
|  | MC-RR | MC-YR | MC-LR | MC-RR | MC-YR | MC-LR |  |  |  |  |
| March | 0.0000 | 0.0050 | 0.1120 | 0.0359 | 0.0110 | 0.0000 | 0.0359 | 0.0159 | 0.1120 | 0.1638 |
| April | 0.0457 | 0.0129 | 0.0451 | 0.0411 | 0.0051 | 0.0000 | 0.0868 | 0.0179 | 0.0451 | 0.1498 |
| May | 0.0168 | 0.0524 | 0.0521 | 0.0000 | 0.0521 | 0.0206 | 0.0168 | 0.1045 | 0.0727 | 0.1940 |
| June | 0.0123 | 0.0000 | 0.0295 | 0.0000 | 0.0195 | 0.0221 | 0.0123 | 0.0195 | 0.0516 | 0.0834 |
| July | 0.1140 | 0.0033 | 0.0146 | 0.0526 | 0.0000 | 0.0000 | 0.1666 | 0.0033 | 0.0146 | 0.1845 |
| August | 0.0704 | 0.0346 | 0.0411 | 0.0804 | 0.0161 | 0.0320 | 0.1509 | 0.0507 | 0.0731 | 0.2747 |
| September | 0.0178 | 0.0368 | 0.0000 | 0.0903 | 0.0149 | 0.0218 | 0.1082 | 0.0517 | 0.0218 | 0.1816 |
| October | 0.0000 | 0.0000 | 0.0598 | 0.0308 | 0.1146 | 0.1125 | 0.0308 | 0.1146 | 0.1722 | 0.3177 |
| November | 0.0000 | 0.0445 | 0.1446 | 0.0246 | 0.0000 | 0.0165 | 0.0246 | 0.0445 | 0.1611 | 0.2302 |
| December | 0.0111 | 0.0112 | 0.0044 | 0.1088 | 0.0044 | 0.0184 | 0.1199 | 0.0156 | 0.0228 | 0.1583 |

**Table 6 The concentration of microcystins from the late March to December in Jiangnan District, Yongjiang River (μg/L)**

| Sampling time | EMCs | | | IMCs | | | T-RR | T-YR | T-LR | TMCs |
| --- | --- | --- | --- | --- | --- | --- | --- | --- | --- | --- |
|  | MC-RR | MC-YR | MC-LR | MC-RR | MC-YR | MC-LR |  |  |  |  |
| March | 0.0000 | 0.0112 | 0.0000 | 0.0452 | 0.0719 | 0.0585 | 0.0452 | 0.0831 | 0.0585 | 0.1868 |
| April | 0.0667 | 0.0107 | 0.0235 | 0.0502 | 0.0000 | 0.0146 | 0.1169 | 0.0107 | 0.0381 | 0.1656 |
| May | 0.0302 | 0.0211 | 0.0222 | 0.0043 | 0.0000 | 0.0344 | 0.0345 | 0.0211 | 0.0566 | 0.1121 |
| June | 0.0346 | 0.0000 | 0.0000 | 0.0066 | 0.1144 | 0.0191 | 0.0412 | 0.1144 | 0.0191 | 0.1746 |
| July | 0.0540 | 0.0000 | 0.0133 | 0.0306 | 0.0038 | 0.0000 | 0.0846 | 0.0038 | 0.0133 | 0.1018 |
| August | 0.0000 | 0.1192 | 0.0668 | 0.0191 | 0.0000 | 0.0000 | 0.0191 | 0.1192 | 0.0668 | 0.2051 |
| September | 0.0960 | 0.0000 | 0.0000 | 0.0401 | 0.0117 | 0.0100 | 0.1361 | 0.0117 | 0.0100 | 0.1578 |
| October | 0.0569 | 0.0192 | 0.1004 | 0.0459 | 0.0000 | 0.0233 | 0.1027 | 0.0192 | 0.1237 | 0.2456 |
| November | 0.0000 | 0.0110 | 0.1457 | 0.0672 | 0.0781 | 0.0000 | 0.0672 | 0.0891 | 0.1457 | 0.3020 |
| December | 0.0842 | 0.0112 | 0.0233 | 0.0000 | 0.0565 | 0.0561 | 0.0842 | 0.0677 | 0.0794 | 0.2313 |

**Table 7 The concentration of microcystins from the beginning of March to December in Yongning District, Yongjiang River (μg/L)**

| Sampling time | EMCs | | | IMCs | | | T-RR | T-YR | T-LR | TMCs |
| --- | --- | --- | --- | --- | --- | --- | --- | --- | --- | --- |
|  | MC-RR | MC-YR | MC-LR | MC-RR | MC-YR | MC-LR |  |  |  |  |
| March | 0.0211 | 0.0079 | 0.0163 | 0.0140 | 0.0000 | 0.0000 | 0.0352 | 0.0079 | 0.0163 | 0.0594 |
| April | 0.0255 | 0.0151 | 0.0126 | 0.0000 | 0.0343 | 0.0064 | 0.0255 | 0.0495 | 0.0189 | 0.0939 |
| May | 0.0000 | 0.0000 | 0.0155 | 0.1103 | 0.0217 | 0.0131 | 0.1103 | 0.0217 | 0.0286 | 0.1606 |
| June | 0.0304 | 0.0000 | 0.0445 | 0.0453 | 0.0207 | 0.0603 | 0.0757 | 0.0207 | 0.1048 | 0.2011 |
| July | 0.0112 | 0.0981 | 0.0450 | 0.0000 | 0.0000 | 0.0559 | 0.0112 | 0.0981 | 0.1009 | 0.2102 |
| August | 0.0000 | 0.0199 | 0.0000 | 0.0145 | 0.0443 | 0.1007 | 0.0145 | 0.0643 | 0.1007 | 0.1794 |
| September | 0.0235 | 0.0107 | 0.0000 | 0.0343 | 0.0508 | 0.1028 | 0.0577 | 0.0615 | 0.1028 | 0.2220 |
| October | 0.0457 | 0.0000 | 0.0043 | 0.0237 | 0.1116 | 0.0000 | 0.0694 | 0.1116 | 0.0043 | 0.1852 |
| November | 0.0000 | 0.0000 | 0.0000 | 0.0237 | 0.0102 | 0.0452 | 0.0237 | 0.0102 | 0.0452 | 0.0792 |
| December | 0.0217 | 0.0062 | 0.0546 | 0.1232 | 0.0000 | 0.0676 | 0.1449 | 0.0062 | 0.1222 | 0.2733 |

**Table 8 The concentration of microcystins from the middle of March to December in Yongning District, Yongjiang River (μg/L)**

| Sampling time | EMCs | | | IMCs | | | T-RR | T-YR | T-LR | TMCs |
| --- | --- | --- | --- | --- | --- | --- | --- | --- | --- | --- |
|  | MC-RR | MC-YR | MC-LR | MC-RR | MC-YR | MC-LR |  |  |  |  |
| March | 0.0185 | 0.0000 | 0.0062 | 0.0000 | 0.0103 | 0.0033 | 0.0185 | 0.0103 | 0.0095 | 0.0384 |
| April | 0.0267 | 0.0233 | 0.0152 | 0.0451 | 0.0000 | 0.0131 | 0.0718 | 0.0233 | 0.0283 | 0.1234 |
| May | 0.0240 | 0.0451 | 0.0000 | 0.0000 | 0.0155 | 0.0603 | 0.0240 | 0.0606 | 0.0603 | 0.1449 |
| June | 0.0146 | 0.0311 | 0.0303 | 0.0474 | 0.0345 | 0.0000 | 0.0620 | 0.0655 | 0.0303 | 0.1578 |
| July | 0.0000 | 0.0000 | 0.0306 | 0.1016 | 0.0310 | 0.0117 | 0.1016 | 0.0310 | 0.0424 | 0.1749 |
| August | 0.0000 | 0.0169 | 0.1661 | 0.0519 | 0.0000 | 0.0128 | 0.0519 | 0.0169 | 0.1789 | 0.2477 |
| September | 0.0060 | 0.0129 | 0.0401 | 0.1217 | 0.0000 | 0.0593 | 0.1277 | 0.0129 | 0.0994 | 0.2400 |
| October | 0.0000 | 0.0342 | 0.0000 | 0.3783 | 0.0000 | 0.0460 | 0.3783 | 0.0342 | 0.0460 | 0.4585 |
| November | 0.0112 | 0.0000 | 0.2002 | 0.1031 | 0.0087 | 0.0066 | 0.1143 | 0.0087 | 0.2069 | 0.3298 |
| December | 0.1142 | 0.0000 | 0.1310 | 0.0000 | 0.0346 | 0.0000 | 0.1142 | 0.0346 | 0.1310 | 0.2798 |

**Table 9 The concentration of microcystins from the late March to December in Yongning District, Yongjiang River (μg/L)**

| Sampling time | EMCs | | | IMCs | | | T-RR | T-YR | T-LR | TMCs |
| --- | --- | --- | --- | --- | --- | --- | --- | --- | --- | --- |
|  | MC-RR | MC-YR | MC-LR | MC-RR | MC-YR | MC-LR |  |  |  |  |
| March | 0.0562 | 0.0000 | 0.0896 | 0.0000 | 0.0231 | 0.0568 | 0.0562 | 0.0231 | 0.1463 | 0.2256 |
| April | 0.0000 | 0.0112 | 0.0345 | 0.0084 | 0.0000 | 0.0000 | 0.0084 | 0.0112 | 0.0345 | 0.0541 |
| May | 0.0402 | 0.0000 | 0.0123 | 0.0310 | 0.0444 | 0.0000 | 0.0712 | 0.0444 | 0.0123 | 0.1279 |
| June | 0.0000 | 0.0000 | 0.0157 | 0.0139 | 0.0343 | 0.0315 | 0.0139 | 0.0343 | 0.0471 | 0.0953 |
| July | 0.0501 | 0.0070 | 0.0000 | 0.0164 | 0.0455 | 0.0322 | 0.0665 | 0.0524 | 0.0322 | 0.1512 |
| August | 0.0000 | 0.0000 | 0.0116 | 0.0565 | 0.0253 | 0.0000 | 0.0565 | 0.0253 | 0.0116 | 0.0934 |
| September | 0.0179 | 0.0345 | 0.0000 | 0.0000 | 0.0404 | 0.0678 | 0.0179 | 0.0748 | 0.0678 | 0.1606 |
| October | 0.0344 | 0.1112 | 0.2210 | 0.0000 | 0.0321 | 0.0452 | 0.0344 | 0.1433 | 0.2663 | 0.4440 |
| November | 0.0318 | 0.0237 | 0.0000 | 0.0189 | 0.0000 | 0.0356 | 0.0508 | 0.0237 | 0.0356 | 0.1101 |
| December | 0.1007 | 0.0213 | 0.0196 | 0.0341 | 0.0000 | 0.0673 | 0.1348 | 0.0213 | 0.0869 | 0.2430 |
