# Supplementary material for: Influencing factors and health risk assessment of microcystins in the Yongjiang river (China) by Monte Carlo simulation
Source: PeerJ. 2018 Nov 16;6:e5955. doi: 10.7717/peerj.5955 (PMC6241391; doi:10.7717/peerj.5955)
Supplement: Supplemental Information 2 — The river section includes three sampling points: Qingxiu District, Jiangnan District, and Yongning District; the environmental factors includes 6 indicators: water temperature, pH, dissolved oxygen, total phosphorus, PO43−-P, and total nitrogen. [file peerj-06-5955-s002.doc]

**Sampling registration form**

| Sampling location | In Qingxiu District, Jiangnan District, YongningDistrict, Nanning, China |
| --- | --- |
| Sampling time | Time: 11:00 to 14:00. The early part of the month is the 5th, the middle part is the 15th, and the late part is the 25th |
| Sampling staff | Chan-Chan Xiao , Mao-Jian Chen, Fan-Biao Mei , Xiang Fang. |

**Table 1 Data of environmental factors from the beginning of March to**

**September in Qingxiu District, Yongjiang River**

| Sampling  time | Water Temperature  (℃) | pH | Total phosphorus  (mg/L) | PO_4_^3-^-P  (mg/L) | Total Nitrogen  (mg/L) | Dissolved Oxygen  (mg/L) | TN:TP  ratio |
| --- | --- | --- | --- | --- | --- | --- | --- |
| March | 17.80 | 7.70 | 0.50 | 0.15 | 15.00 | 6.50 | 30.00 |
| April | 23.80 | 7.80 | 0.80 | 0.35 | 11.00 | 6.00 | 13.75 |
| May | 23.60 | 7.80 | 2.50 | 1.15 | 5.00 | 3.50 | 2.00 |
| June | 24.50 | 7.50 | 2.25 | 0.75 | 1.50 | 3.30 | 0.67 |
| July | 27.60 | 6.80 | 4.20 | 1.35 | 1.50 | 2.20 | 0.36 |
| August | 27.10 | 6.70 | 2.10 | 0.50 | 12.00 | 3.00 | 5.71 |
| September | 28.70 | 6.80 | 2.20 | 0.55 | 2.50 | 2.50 | 1.14 |
| October | 26.70 | 6.50 | 1.50 | 0.50 | 1.50 | 3.60 | 1.00 |
| November | 27.40 | 6.90 | 3.60 | 0.75 | 8.00 | 2.60 | 2.22 |
| December | 26.90 | 7.80 | 2.65 | 0.65 | 2.50 | 2.60 | 0.94 |

**Table 2 Data of environmental factors from the middle of March to September in Qingxiu District, Yongjiang River**

| Sampling  time | Water Temperature  (℃) | pH | Total phosphorus  (mg/L) | PO_4_^3-^-P  (mg/L) | Total Nitrogen  (mg/L) | Dissolved Oxygen  (mg/L) | TN:TP  ratio |
| --- | --- | --- | --- | --- | --- | --- | --- |
| March | 17.60 | 7.80 | 0.45 | 0.25 | 4.50 | 9.00 | 10.00 |
| April | 23.30 | 7.60 | 2.35 | 0.75 | 1.00 | 2.40 | 0.43 |
| May | 15.90 | 7.80 | 0.15 | 0.11 | 2.50 | 9.00 | 16.67 |
| June | 16.70 | 7.80 | 0.35 | 0.05 | 1.50 | 6.40 | 4.29 |
| July | 17.50 | 7.90 | 0.15 | 0.12 | 4.50 | 9.00 | 30.00 |
| August | 26.80 | 7.40 | 2.18 | 0.72 | 5.00 | 2.50 | 2.29 |
| September | 26.60 | 7.10 | 1.50 | 0.50 | 10.00 | 3.50 | 6.67 |
| October | 24.40 | 6.50 | 2.15 | 0.50 | 2.00 | 2.30 | 0.93 |
| November | 26.30 | 7.70 | 3.15 | 0.85 | 3.00 | 2.00 | 0.95 |
| December | 15.40 | 6.80 | 0.45 | 0.15 | 3.00 | 4.50 | 6.67 |

**Table 3 Data of environmental factors from the late March to September in Qingxiu District, Yongjiang River**

| Sampling  time | Water Temperature  (℃) | pH | Total phosphorus  (mg/L) | PO_4_^3-^-P  (mg/L) | Total Nitrogen  (mg/L) | Dissolved Oxygen  (mg/L) | TN:TP  ratio |
| --- | --- | --- | --- | --- | --- | --- | --- |
| March | 25.60 | 7.40 | 0.75 | 0.35 | 9.00 | 6.40 | 12.00 |
| April | 18.60 | 7.60 | 0.25 | 0.15 | 2.50 | 7.40 | 10.00 |
| May | 16.40 | 7.80 | 0.35 | 0.45 | 11.00 | 7.50 | 31.43 |
| June | 23.50 | 7.10 | 0.28 | 0.50 | 7.50 | 12.00 | 26.79 |
| July | 22.70 | 7.40 | 0.25 | 0.10 | 12.00 | 10.00 | 48.00 |
| August | 24.40 | 7.60 | 0.20 | 0.10 | 1.70 | 3.20 | 8.50 |
| September | 26.40 | 7.30 | 3.25 | 0.75 | 1.50 | 2.20 | 0.46 |
| October | 26.90 | 6.90 | 2.15 | 0.43 | 2.50 | 3.40 | 1.16 |
| November | 26.70 | 7.80 | 0.70 | 0.50 | 2.00 | 8.50 | 2.86 |
| December | 26.50 | 7.40 | 0.35 | 0.25 | 2.00 | 11.00 | 5.71 |

**Table 4 Data of environmental factors from the beginning of March to Septemberin Jiangnan District, Yongjiang River**

| Sampling  time | Water Temperature  (℃) | pH | Total phosphorus  (mg/L) | PO_4_^3-^-P  (mg/L) | Total Nitrogen  (mg/L) | Dissolved Oxygen  (mg/L) | TN:TP  ratio |
| --- | --- | --- | --- | --- | --- | --- | --- |
| March | 20.40 | 7.50 | 0.70 | 0.25 | 1.50 | 8.00 | 2.14 |
| April | 15.80 | 7.70 | 0.65 | 0.35 | 1.50 | 7.00 | 2.31 |
| May | 21.50 | 6.60 | 0.25 | 0.15 | 1.50 | 8.00 | 6.00 |
| June | 26.40 | 7.30 | 1.65 | 0.15 | 1.00 | 3.66 | 0.61 |
| July | 17.40 | 7.80 | 0.25 | 0.15 | 1.00 | 11.00 | 4.00 |
| August | 22.10 | 7.20 | 0.70 | 0.45 | 16.00 | 9.40 | 22.86 |
| September | 26.40 | 6.70 | 1.15 | 0.05 | 2.50 | 7.60 | 2.17 |
| October | 26.80 | 6.80 | 1.50 | 0.35 | 12.00 | 3.60 | 8.00 |
| November | 27.10 | 6.60 | 0.60 | 0.25 | 13.00 | 6.00 | 21.67 |
| December | 21.70 | 7.80 | 1.30 | 1.15 | 4.00 | 8.00 | 3.08 |

**Table 5 Data of environmental factors from the middle of March to Septemberin Jiangnan District, Yongjiang River**

| Sampling  time | Water Temperature  (℃) | pH | Total phosphorus  (mg/L) | PO_4_^3-^-P  (mg/L) | Total Nitrogen  (mg/L) | Dissolved Oxygen  (mg/L) | TN:TP  ratio |
| --- | --- | --- | --- | --- | --- | --- | --- |
| March | 26.10 | 7.50 | 3.20 | 0.75 | 4.50 | 3.60 | 1.41 |
| April | 26.30 | 7.50 | 1.20 | 0.70 | 1.50 | 7.00 | 1.25 |
| May | 25.40 | 7.80 | 2.30 | 0.35 | 6.00 | 5.60 | 2.61 |
| June | 23.10 | 7.80 | 1.45 | 0.35 | 1.50 | 6.40 | 1.03 |
| July | 25.50 | 6.70 | 0.85 | 0.45 | 2.00 | 8.00 | 2.35 |
| August | 26.60 | 7.70 | 1.25 | 0.30 | 2.00 | 6.80 | 1.60 |
| September | 25.60 | 7.50 | 0.23 | 0.05 | 1.60 | 8.60 | 6.96 |
| October | 24.50 | 7.80 | 3.75 | 1.35 | 4.00 | 3.00 | 1.07 |
| November | 23.40 | 7.70 | 3.50 | 0.75 | 2.50 | 2.40 | 0.71 |
| December | 24.60 | 7.00 | 0.40 | 0.25 | 3.50 | 6.00 | 8.75 |

**Table 6 Data of environmental factors from the late March to Septemberin Jiangnan District, Yongjiang River**

| Sampling  time | Water Temperature  (℃) | pH | Total phosphorus  (mg/L) | PO_4_^3-^-P  (mg/L) | Total Nitrogen  (mg/L) | Dissolved Oxygen  (mg/L) | TN:TP  ratio |
| --- | --- | --- | --- | --- | --- | --- | --- |
| March | 26.30 | 7.60 | 0.35 | 0.10 | 1.50 | 4.20 | 4.29 |
| April | 25.70 | 6.90 | 0.35 | 0.20 | 3.50 | 8.00 | 10.00 |
| May | 24.30 | 7.80 | 0.43 | 0.10 | 1.40 | 5.80 | 3.26 |
| June | 24.20 | 7.40 | 0.45 | 0.15 | 1.50 | 11.00 | 3.33 |
| July | 25.20 | 7.30 | 0.15 | 0.45 | 2.50 | 8.00 | 16.67 |
| August | 26.30 | 7.70 | 1.20 | 0.55 | 2.50 | 2.60 | 2.08 |
| September | 25.40 | 6.70 | 0.34 | 0.15 | 11.00 | 6.00 | 32.35 |
| October | 26.40 | 6.80 | 3.15 | 0.25 | 1.50 | 2.50 | 0.48 |
| November | 26.40 | 7.30 | 3.15 | 0.15 | 1.50 | 2.80 | 0.48 |
| December | 26.90 | 7.70 | 1.15 | 0.01 | 12.00 | 3.40 | 10.43 |

**Table 7 Data of environmental factors from the beginning of March to Septemberin Yongning District, Yongjiang River**

| Sampling  time | Water Temperature  (℃) | pH | Total phosphorus  (mg/L) | PO_4_^3-^-P  (mg/L) | Total Nitrogen  (mg/L) | Dissolved Oxygen  (mg/L) | TN:TP  ratio |
| --- | --- | --- | --- | --- | --- | --- | --- |
| March | 20.70 | 7.70 | 0.75 | 0.05 | 2.50 | 9.00 | 3.33 |
| April | 21.50 | 7.60 | 0.25 | 0.15 | 1.50 | 12.50 | 6.00 |
| May | 25.40 | 6.80 | 2.75 | 0.12 | 8.00 | 9.50 | 2.91 |
| June | 26.80 | 7.80 | 2.80 | 0.25 | 2.50 | 4.30 | 0.89 |
| July | 26.50 | 7.50 | 3.25 | 0.10 | 4.50 | 2.50 | 1.38 |
| August | 26.10 | 7.50 | 3.20 | 0.15 | 1.00 | 2.60 | 0.31 |
| September | 26.40 | 7.10 | 2.25 | 0.05 | 2.50 | 2.00 | 1.11 |
| October | 26.10 | 7.60 | 1.15 | 0.35 | 1.50 | 12.00 | 1.30 |
| November | 25.40 | 7.80 | 0.70 | 0.15 | 4.50 | 4.00 | 6.43 |
| December | 26.80 | 6.70 | 2.35 | 0.60 | 5.00 | 2.60 | 2.13 |

**Table 8 Data of environmental factors from the middle of March to Septemberin Yongning District, Yongjiang River**

| Sampling  time | Water Temperature  (℃) | pH | Total phosphorus  (mg/L) | PO_4_^3-^-P  (mg/L) | Total Nitrogen  (mg/L) | Dissolved Oxygen  (mg/L) | TN:TP  ratio |
| --- | --- | --- | --- | --- | --- | --- | --- |
| March | 17.10 | 7.50 | 0.70 | 0.50 | 10.00 | 10.00 | 14.29 |
| April | 24.70 | 7.80 | 1.60 | 0.19 | 2.00 | 8.00 | 1.25 |
| May | 23.10 | 7.50 | 0.25 | 0.15 | 3.00 | 3.80 | 12.00 |
| June | 21.30 | 7.50 | 0.30 | 0.25 | 3.00 | 8.00 | 10.00 |
| July | 23.40 | 7.30 | 0.25 | 0.13 | 9.00 | 8.50 | 36.00 |
| August | 25.20 | 7.50 | 3.70 | 0.50 | 2.50 | 2.10 | 0.68 |
| September | 25.50 | 6.80 | 2.15 | 0.25 | 11.00 | 4.00 | 5.12 |
| October | 24.60 | 6.50 | 0.85 | 0.15 | 7.50 | 5.00 | 8.82 |
| November | 27.60 | 6.80 | 3.60 | 0.45 | 12.00 | 4.00 | 3.33 |
| December | 27.50 | 7.60 | 3.30 | 0.15 | 1.70 | 6.00 | 0.52 |

**Table 9 Data of environmental factors from the late March to September in Yongning District, Yongjiang River**

| Sampling  time | Water Temperature  (℃) | pH | Total phosphorus  (mg/L) | PO_4_^3-^-P  (mg/L) | Total Nitrogen  (mg/L) | Dissolved Oxygen  (mg/L) | TN:TP  ratio |
| --- | --- | --- | --- | --- | --- | --- | --- |
| March | 26.40 | 7.50 | 2.20 | 0.20 | 1.50 | 2.80 | 0.68 |
| April | 18.60 | 7.80 | 0.42 | 0.15 | 2.50 | 4.00 | 5.95 |
| May | 25.30 | 7.40 | 0.23 | 0.45 | 2.00 | 9.00 | 8.70 |
| June | 19.40 | 7.90 | 0.45 | 0.15 | 2.00 | 3.60 | 4.44 |
| July | 23.30 | 6.70 | 0.85 | 1.15 | 1.50 | 6.00 | 1.76 |
| August | 22.20 | 7.70 | 0.50 | 0.45 | 1.50 | 8.00 | 3.00 |
| September | 25.20 | 7.60 | 0.23 | 0.15 | 1.50 | 6.00 | 6.52 |
| October | 24.30 | 7.20 | 3.75 | 0.55 | 1.00 | 2.60 | 0.27 |
| November | 25.40 | 6.80 | 0.75 | 0.70 | 1.00 | 8.60 | 1.33 |
| December | 26.10 | 6.90 | 1.50 | 0.15 | 16.00 | 4.60 | 10.67 |
